# Response of cultured primary gingival and periodontal ligament cells to angiotensin II and IL1β challenges

**DOI:** 10.1590/1807-3107bor-2025.vol39.083

**Published:** 2025-09-08

**Authors:** Thais Francini GARBIERI, Thiago José DIONÍSIO, Bella Luna COLOMBINI-ISHIKIRIAMA, Rafaela Alves da SILVA, Vanessa Soares LARA, Sandra Helena Penha OLIVEIRA, Maria Helena FERNANDES, Andrew Seth Greene, Carlos Ferreira SANTOS

**Affiliations:** (a) Universidade de São Paulo – USP, Bauru School of Dentistry, Department of Biological Sciences, Bauru, SP, Brazil.; (b) Universidade de São Paulo – USP, Bauru School of Dentistry, Department of Surgery, Stomatology, Pathology and Radiology, Bauru, SP, Brazil.; (c) Universidade Estadual Paulista – Unesp, Araçatuba School of Dentistry, Department of Basic Sciences, Araçatuba, SP, Brazil.; (d) Universidade do Porto, School of Dental Medicine, Laboratory for Bone Metabolism and Regeneration, Porto, Portugal.; (e) The Jackson Laboratory, Bar Harbor, ME, USA.

**Keywords:** Renin-Angiotensin System, Angiotensin, Primary Cell Culture, Periodontium

## Abstract

Angiotensin II (Ang II) releases inflammatory mediators from several cell types. The objective of this study was to investigate the potential of Ang II to induce mRNA expression of inflammatory mediators in primary cultured fibroblast-like cells isolated from gingival and periodontal ligament tissues. A synergistic effect of co-treatment with Ang II and Interleukin-1β (IL1β) on the mRNA expression of inflammatory mediators was explored. Immunophenotyping of STRO-1, Ang II type 1 receptor (AT1R), and Ang II type 2 receptor (AT2R) was performed using flow cytometry. Cell cultures were challenged with Ang II (1 µM) for 3, 6, and 24 h with or without co-treatment with IL1β (0.1 ng/mL) for 24 h. mRNA expression of inflammatory mediators was determined using qPCR. We present, for the first time, precise quantification of AT1R and AT2R in human gingival and periodontal fibroblast-like cell types; the percentage of positive immunostaining compared to the total cell population varied from 3.35% to 5.29% for AT1R and 2.97% to 4.57% for AT2R. Ang II slightly upregulated IL6 and CCL2/MCP1 mRNA expression in gingival cells and IL8 and PTGS2/COX2 in periodontal ligament cells. IL1β upregulated IL8, IL6, CCL2/MCP1, PTGS2/COX2, and IL1β mRNA in both cell types. Co-treatment with Ang II and IL1β did not show a synergistic effect. Ang II showed a low potential to induce mRNA of inflammatory mediators, most likely owing to the low percentage of Ang II receptors in such cells and no synergistic effect with the co-treatment with IL1β.

## Introduction

The renin-angiotensin system (RAS) functions locally in various tissues, collectively referred to as the local RAS or the tissue renin-angiotensin system (tRAS), while regulating several essential functions of the body, such as those related to systemic blood pressure and cardiovascular homeostasis.^1,2^ Locally, the main functions of these systems are related to inflammation, aging, cell proliferation, and fibrosis, mainly through the modulation of the primary active mediator of the system, angiotensin II (Ang II), which exerts its effects through interactions with specific AT1 and AT2 G protein-coupled receptors.^1,3^ Several studies have associated the involvement of the tRAS and local RAS with periodontal pathology, suggesting an impact on periodontitis and bone loss caused by an imbalance of periodontal tissue.^3-6^ In rats, Angiotensin Receptor Type 1 (AT1R) blockade by losartan modulates experimental periodontitis because it reduces the mRNA expression of proinflammatory mediators and osteoclastogenesis, reduces bone resorption, and prevents bone loss. Bone formation was affected by Ang II acting via AT1R in an ex vivo model using an embryonic chick femur organotypic culture, in which negative effects of Ang II on bone formation were observed.^7^

Regarding the role of Ang II/AT1R axis on inflammation, several cell types (both animal and human), such as smooth muscle cells, THP-1 monocytes, bone marrow mesenchymal stem cells, mesangial cells, adipocytes, pancreatic islet, cardiac and lung fibroblasts, and vascular smooth muscle cells, respond to an Ang II challenge, expressing and/or producing proinflammatory mediators such as interleukin (IL)18, IL6, IL1β, C-X-C motif chemokine ligand 8 (CXCL8 or IL8), tumor necrosis factor α (TNFα), monocyte chemoattractant protein-1 (MCP1)/C-C motif chemokine ligand 2 (CCL2), cyclooxygenase-2 (COX2)/prostaglandin-endoperoxide synthase 2 (PTGS2), and prostaglandin E2 (PGE2).^8-15^. Ang II is upregulated in the periodontal disease of rats with primary hypertension, directly and indirectly increasing the inflammatory response.^16^

Oral tissue cells produce several inflammatory mediators when challenged with various stimuli. When added to primary cultured oral fibroblasts, IL1β upregulates IL6, IL1β, IL8, and TNFα,^5,17^ induces AT1R expression in different cell types,^18,19^ and is an important cytokine in periodontal disease.

The rationale for gingival fibroblast/PDL cell types selection, and a possible synergism between the Ang II/ATR axis and IL1β signaling in inflammatory mediators expression, was based on the role of these cell types in inflammation-induced tissue destruction in periodontitis. Therefore, it was considered important to evaluate the capacity of IL1β to upregulate AT1 receptors in these cells by inducing the expression of AT1R in different cell types.^5^ We previously showed that greater AT1R fluorescence intensity occurred in Human Gingival Fibroblast (HGF) and Human Periodontal Ligament Fibroblast (HPLF) stimulated by IL1β compared to stimuli of bacterial origin;^4^ we considered an IL1β stimulus before an Ang II stimulus.

Studies have shown the synergistic induction of COX-2 in pulmonary fibroblasts and MCP1 and IL6 in mesangial cells between Ang II and proinflammatory cytokines such as IL1β.^20,21^ When human oral fibroblasts were challenged by bacterial stimuli such as Porphyromonas gingivalis, lipopolysaccharide, Escherichia coli lipopolysaccharide or Enterococcus faecalis lipoteichoic acid, inflammatory mediators such as macrophage inflammatory protein 1 alpha/C-C motif chemokine ligand 3, stromal-derived factor 1, CXCL12, IL6, IL1α, IL1β, IL8, CCL2, CCL5, TNFα, and colony-stimulating factor 1 were produced.^22-24^ We hypothesized that Ang II has the potential to induce the mRNA expression of inflammatory mediators in cultured primary human gingival and periodontal ligament cells because of the ability of primary cultured oral cells to produce several inflammatory mediators and the capacity of Ang II to induce an inflammatory response in diverse cell types.

## Methods

### Primary cell culture

Primary cultures of human fibroblast-like cells from gingival and periodontal ligament tissues of three healthy adult participants (two females and one male; age range–22–25 years) were established using the explant technique^22-24^ following third molar extraction surgery. This study was approved by the Human Subjects Ethics Board of the Ethics Committee for Human Research of Bauru School of Dentistry, University of São Paulo (CAAE: 77365617.3.0000.5417). Tissues were removed and cultured under aseptic conditions. After fragmentation, tissues were incubated for cell growth in Dulbecco’s modified Eagle’s medium (Gibco™), supplemented with 10% fetal bovine serum (FBS) (Gibco™) and antibiotics (100 U/mL penicillin, 100 mg/mL streptomycin, 0.25 mg/mL amphotericin B, and 0.5 mg/mL gentamicin) (Gibco™). Cultures were maintained at 37ºC in a humidified atmosphere of 5% CO_2_ and 95% air, and the culture medium was changed every 2–3 days. The cells were used between passages four and eight.

### Characterization of primary cell cultures

Primary gingival and periodontal ligament cells were characterized as fibroblasts based on their morphology and positive staining for fibroblast surface protein (FSP) using immunofluorescence techniques as previously described.^5,23-26^ Immunophenotyping using anti-STRO-1, anti-AT1R, and anti-AT2R (Santa Cruz Biotechnology, Dallas, EUA, Cat. No. sc-47733, sc-515884, and sc-518054, respectively) was performed using flow cytometry in a BD FACSAria™ Fusion Cell Sorter (BD Bioscience, San Jose, United States), and data were analyzed using FlowJo™ software. Briefly, cells were maintained under basal conditions and detached using TrypLE™ Express Enzyme (1X) (Gibco™). After counting, 10^6^of each type of cells were separated and processed. Human BD Fc Block™ was used for blocking nonspecific binding sites. Cells were stained with the specific antibodies mentioned above (1:100) for 30 min at 4°C in the dark. Unstained cells were used as negative controls.

### Gene expression analysis

Cellular viability and cytotoxicity against the challenges were analyzed using AlamarBlue® Cell Viability reagent (Invitrogen™, Ambion, Thermo Fisher Scientific, Waltham, USA) following the manufacturer’s instructions.

For gene expression analysis, cells were seeded at 2 × 10^5^ cells/well in 6-well plates in triplicate in basal culture medium. After overnight cell attachment, the culture medium was replaced with reduced FBS (1%). After 24 h, Ang II (1 µM, Sigma-Aldrich^®^) was added to the cells for 3, 6, and 24 h or IL1β (0.1 ng/mL, PeproTech^®^) for 24 h alone or with additional challenge with Ang II for 3, 6, and 24 h. After the experimental period, the supernatants were removed, and the cells were lysed for RNA extraction.

Total RNA was obtained directly from cells using the PureLink RNA Mini Kit (Invitrogen) according to the manufacturer’s instructions. RNA concentration measurement and quality assessment were performed in a spectrophotometer NanoDrop™ 1000 (Thermo Fisher Scientific). cDNA was synthesized using the High-Capacity cDNA Reverse Transcription Kit (Applied Biosystems). Real-time quantitative polymerase chain reaction (RT-qPCR) was performed using a gene expression assay and proprietary primers Taqman™ Gene Expression PCR Master Mix (Applied Biosystems™) targeting mRNA for CXCL8/IL8 (Hs00174103_m1), CCL2/MCP1 (Hs00234140_m1), PTGS2/COX2 (Hs00153133_m1), IL6 (Hs00174131_m1), TNFa (Hs00174128_m1), IL1β (Hs01555410_m1), and AGTR1 (Hs99999095_m1). RPL13A (Hs03043885_g1) was used as a reference gene. All experiments were performed in the ViiA™ 7 Real-Time PCR System (Applied Biosystems™) using the comparative cycle threshold (Ct) method (∆∆Ct) as previously described.^4^

### Statistical analysis

Statistical analyses were performed using the GraphPad Prism 9 software (GraphPad Software, LLC. San Diego, USA). Data were tested for normal distribution using the Shapiro–Wilk test. When data were parametric, an unpaired t-test was used; when data were non-parametric, the Mann–Whitney U test was used to observe the differences between the challenged group and the respective control in the same period of evaluation. Data were expressed as means or medians and analyzed using one-way Analysis of Variance (ANOVA) followed by Tukey’s post-hoc test for multiple comparisons of parametric data and by Kruskal–Wallis test followed by Dunn’s post-hoc test for non-parametric data. Differences were identified at a significance level of 95% (p < 0.05).

## Results

### Characterization of primary cells

**Figure 1:**
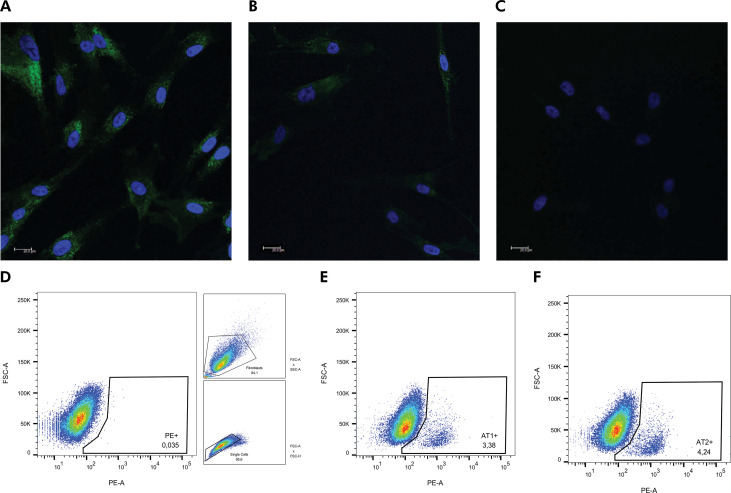
Phenotypic characterization of cultured primary periodontal cells. Cells were plated (104 cells/well) and showed positive staining for FSP-1 protein in green (A - cells from gingival tissue and B - cells from periodontal ligament tissue) through immunofluorescence analysis. Cell nuclei were stained with DAPI-blue (4’,6- diamidino-2- phenylindole dihydrochloride) C - negative control. Images were captured using a confocal microscope (TCS model, SPE, Leica®, Mannheim, Germany). Scale bars - 20 μm. AT1R and AT2R expression by cells considering the whole population. D - Strategy gate to define the stained cells compared to non-stained cells. Percentage of stained cells for AT1R (E) and AT2R (F).

Positivity for FSP in immunofluorescence analysis indicated a fibroblastic phenotype (Figures 1A, 1B, 1C). In the primary cell culture used in this study, it was possible to observe low expression of the STRO-1 marker, varying from 0.013% to 0.69%, which was associated with FSP immunostaining, cell morphology, and specific culture conditions characterizing such cells as fibroblasts. Ang II, AT1R, and AT2R expression levels are shown in Figures 1D, 1E, and 1F. A small percentage of cells expressed these receptors, varying from 3.35% to 5.29% for AT1R and 2.97% to 4.57% for AT2R in the general population.

### mRNA expression of inflammatory mediators in primary human cells from periodontal tissues

#### Ang II slightly increased IL6 and CCL2/MCP1 expression in gingival primary cells and IL8 and PTGS2/COX2 in periodontal ligament cells

**Figure 2:**
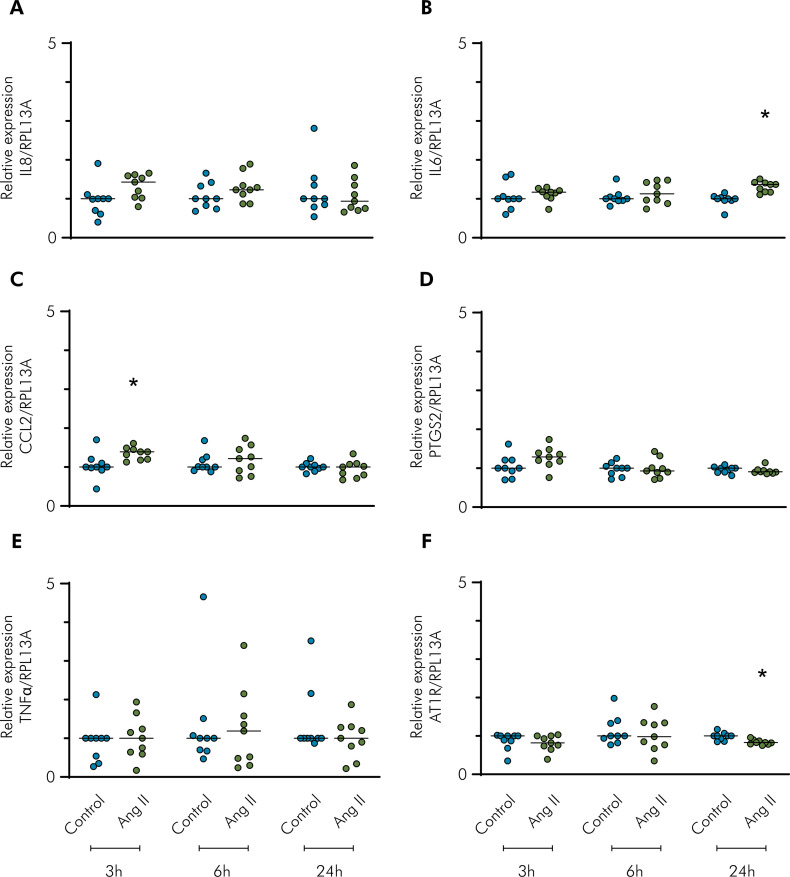
Gene expression analysis using RT-qPCR of inflammatory mediators and AT1R with Ang II challenge. Relative expression levels of the target mRNA to RPL13A mRNA from three donors in triplicate are displayed in graphs. Primary gingival cells were challenged by Ang II (1 µM) for 3, 6, and 24 h, and RT-qPCR analysis was performed for the inflammatory mediators: IL8 (A), IL6 (B), CCL2 (C), PTGS2 (D), TNFα (E), and AT1R (F). The control group corresponds to cells in the basal medium. *Indicates a significant difference in relation to the respective control in the same experimental period (p < 0.05).

**Figure 3:**
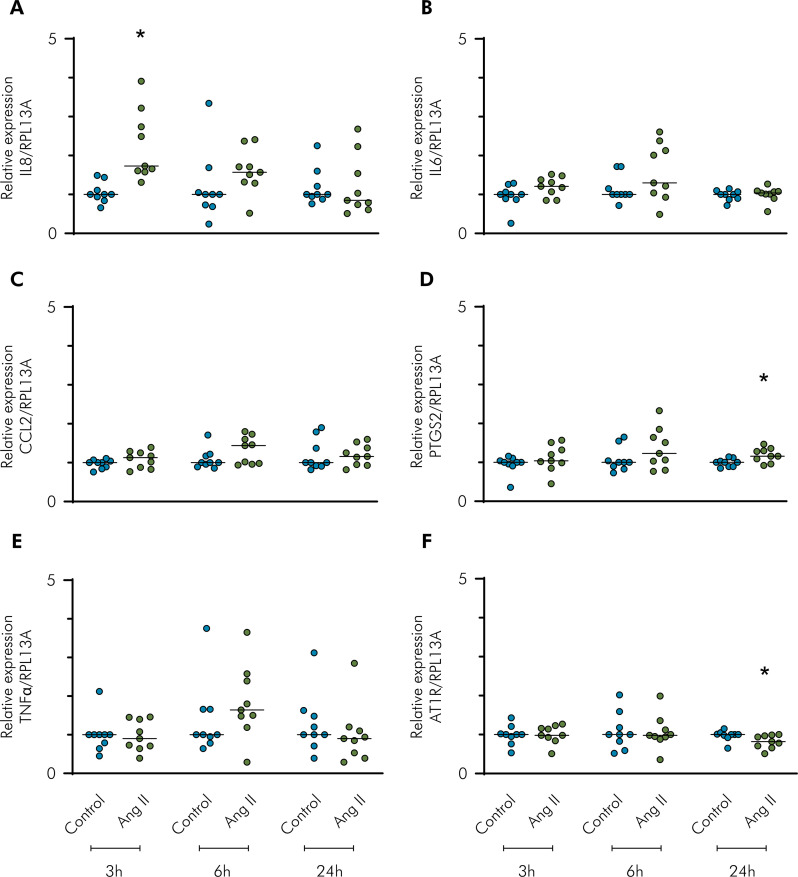
Gene expression analysis using RT-qPCR of inflammatory mediators and AT1R with Ang II challenge. Relative expression levels of the target mRNA to RPL13A mRNA from three donors in triplicate are displayed in graphs. Primary periodontal ligament cells were challenged by Ang II (1 µM) for 3, 6, and 24 h, and RT-qPCR analysis was performed for the inflammatory mediators: IL8 (A), IL6 (B), CCL2 (C), PTGS2 (D), TNFα (E), and AT1R (F). The control group corresponds to cells in the basal medium. * Indicates a significant difference in relation to the respective control in the same experimental period (p < 0.05).

The mRNA expression of inflammatory mediators in the oral cells after Ang II challenge at 3, 6, and 24 h is shown in Figure 2 (gingival cells) and 3 (periodontal ligament cells). At time points of 24 h and 3 h, Ang II challenge slightly upregulated the mRNA expression of IL6 and CCL2, respectively (Figures 2B and 2C). With the addition of Ang II, the mRNA expression of IL8 and PTGS2 was observed at time points of 3 h and 24 h, respectively (Figures 3A and 3D).

#### IL1β for 24 h induced the mRNA of several inflammatory mediators in gingival and periodontal ligament cells, and Ang II did not cause a synergistic effect

**Figure 4:**
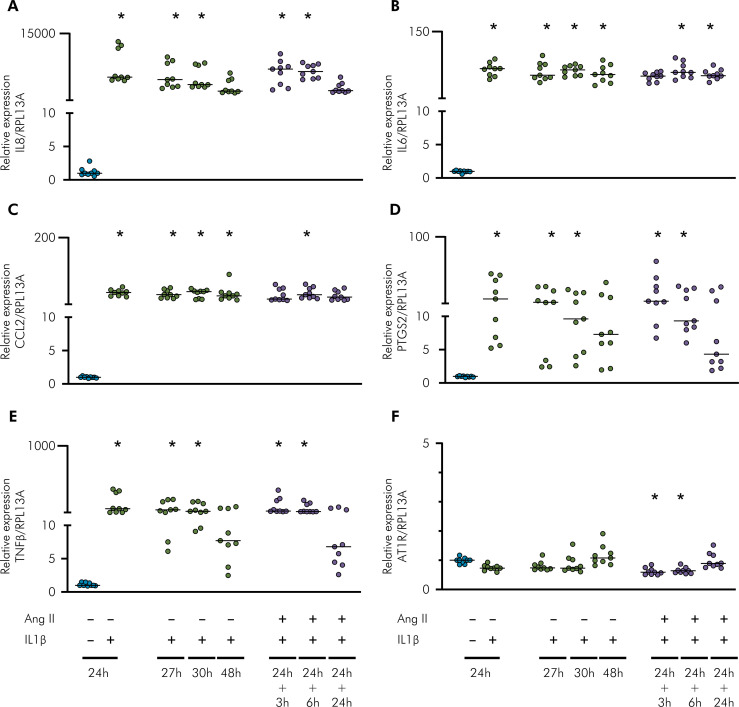
Gene expression analysis using RT-qPCR of inflammatory mediators and AT1R with IL1β and Ang II challenges. Relative expression levels of the target mRNA to RPL13A mRNA from three donors in triplicate are displayed in graphs. Primary gingival cells were challenged by IL1β (0.1 ng/mL) for 24, 27, 30, and 48 h or IL1β for 24 h followed by Ang II (1 µM) for 3, 6, and 24 h. RT-qPCR analysis was performed for the following inflammatory mediators: IL8 (A), IL6 (B), CCL2 (C), PTGS2 (D), IL1β (E), and AT1R (F). An IL1β challenge alone for 27, 30, and 48 h was used to compare with those groups that were challenged with IL1β followed by Ang II for 3, 6, and 24 h, respectively. * Indicates significant difference in comparison to the control (basal medium) (p < 0.05).

**Figure 5:**
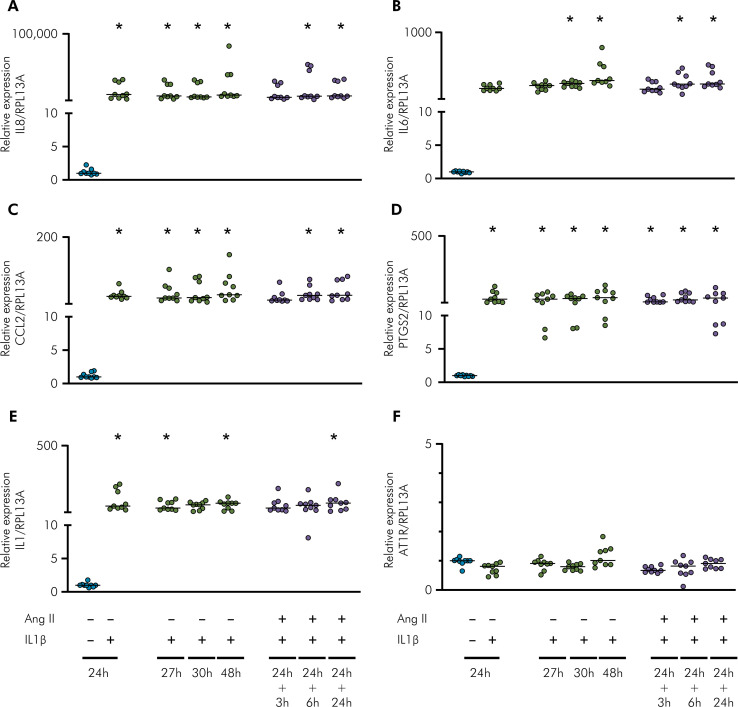
Gene expression analysis using RT-qPCR of inflammatory mediators and AT1R with IL1β and Ang II challenges. Relative expression levels of the target mRNA to RPL13A mRNA from three donors in triplicate are displayed in graphs. Primary periodontal ligament cells were challenged by IL1β (0.1 ng/mL) for 24, 27, 30, and 48 h or IL1β for 24 h followed by Ang II (1 µM) for 3, 6, and 24 h. RT-qPCR analysis was performed for the following inflammatory mediators: IL8 (A), IL6 (B), CCL2 (C), PTGS2 (D), IL1β (E), and AT1R (F). IL1β challenge alone for 27, 30, and 48 h was used to compare with those groups that were challenged with IL1β followed by Ang II for 3, 6, and 24 h, respectively. * Indicates significant difference in comparison to the control (basal medium) (p < 0.05).

In both gingival and periodontal ligament cells, the IL1β challenge was capable of inducing the mRNA of several inflammatory mediators, including IL8, IL6, CCL2, PTGS2, and IL1β (Figure 4, gingival cells; Figure 5, ligament periodontal cells), and Ang II did not have a synergistic effect on IL1β challenge at any tested period.

## Discussion

The Ang II-induced inflammatory functions are primarily linked to AT1R in various cells and tissues.^8,9,27^ Oral cells, mainly fibroblasts, play an important role in the inflammatory response to various stimuli. These cells show positive fluorescent immunostaining for AT1R.^4,28^ Therefore, we hypothesized that Ang II, mediated through its action on AT1R of fibroblasts from oral tissues, modulated immunoinflammatory processes in oral pathologies such as gingivitis and periodontitis.

Gingival and periodontal ligament fibroblasts express AT1R;^4,17^ however, these receptors have never been directly quantified. To the best of our knowledge, we present, for the first time, the precise quantification of AT1R and AT2R in such cell types through immunostaining and flow cytometry. The percentages varied from 3.35% to 5.29% for AT1R and 2.97% to 4.57% for AT2R. Although quantification was not performed for other types of oral cells, such as stem cells of the apical papilla, they present slightly positive immunostaining for AT1R, indicating a low abundance of AT1R.^29^ In addition, we must consider the presence of nuclear AT1R and the intracellular effects of Ang II.^17,30^

We only evaluated fibroblast-like cells, but living organisms have other cells in the periodontal tissue, which contain AT1R and AT2R^4^ and may play a role in the response to Ang II. Emphasizing the anti-inflammatory effects of AT1R antagonism by losartan, which decreases inflammation and bone loss in the periodontal tissues of rats with experimentally induced periodontal disease, is important.^6^ Although slight immunostaining for AT1R and AT2R was observed in the cell cultures in the current study, the importance of such receptors cannot be disregarded in the whole animal because other cells of the periodontal tissue may interact to Ang II. Moreover, for future studies, we consider the possibility of separating and conducting the experiments only with cells positive for AT1R and AT2R. A pilot study was conducted to isolate only AT1R- and AT2R-marked cells for cultivation. However, because of the low number of recovered cells, the establishment of the cultures was not successful. In addition, the systemic effects of Ang II binding to its receptors should always be considered in the entire organism.

Primary gingival and periodontal ligament cells were characterized as fibroblasts by their morphology and positive staining for FSP using an immunofluorescence technique, as previously described.^5,23-26^ However, mesenchymal stromal cells/stem cells (MSCs), which can be isolated from human dental tissues, show a fibroblast-like morphology and express the same surface markers, such as FSP, being considered phenotypically indistinguishable from fibroblasts.^31^ In the current study, we used the distinct marker STRO-1, which is more highly expressed in MSCs than in fibroblasts, to distinguish between these cell types.^32^ Therefore, we concluded that the primary cultures used in this study were fibroblast-like oral cells with a low, variable proportion of MSCs.

The inflammatory mediators analyzed in this study were selected based on a previous study that detected the modulatory role of Ang II in fibroblasts from the kidney, heart, and lung.^14,20,33^ These mediators are important in periodontal pathology and inflammatory diseases. CXCL8/IL8 and CCL2/MCP1 are chemokines that are of great importance in the inflammatory process because of their ability to attract neutrophils and monocytes, respectively, to the site of inflammation.^34,35^ IL6 is an important proinflammatory cytokine that affects osteoclastogenesis and bone resorption.^5^ PTGS2 can be induced by cytokines during inflammation and is responsible for the elevated production of prostaglandins, which are potent inflammatory mediators.^14,20^ Rat cardiac fibroblasts challenged with Ang II showed intense expression of COX2 protein in a time-dependent manner, which correlates with a significant increase in prostaglandin E2 release.^14^ Ang II induces prostaglandin E2 release in human gingival fibroblasts^27^ and COX2 expression in mouse lung fibroblasts but not in human lung adenocarcinoma (A549) or normal human bronchial epithelial cells.^20^ In the current study, Ang II was not capable of inducing PTGS2 expression in gingival fibroblasts; however, a slight upregulation was observed in cells derived from the periodontal ligament at the 24 h time point.

The mRNA expression of AT1R was downregulated by the addition of Ang II at the 24 h time point in both cell types. IL1β alone (0.1 ng/mL) did not induce a slight increase in AT1R, in contrast to the findings of other studies.^18,19^ These results suggest that fibroblasts originating from different compartments of periodontal tissue (gingiva and periodontal ligament) have different abilities to regulate AT1R mRNA expression. IL1β was able to significantly increase mRNA expression of several inflammatory mediators with great importance for periodontal pathologies, such as IL8, PTGS2, CCL2, IL6, and IL1β, when compared to the non-challenged group of cells. Measuring the proteins for any cytokine that is significantly altered at the RNA level is important. This is a limitation of the current work.

Although there are reports documenting the synergism between IL1β and Ang II, increasing COX2 production in lung fibroblasts^20^ and MCP1 and IL6 in mesangial cells,^21^ no such interaction was observed in the current study, suggesting that the intracellular mechanisms facilitating such a molecular interaction are not present in these cells.

## Conclusions

Ang II challenge of cultured primary human gingival and periodontal ligament cells induced only a slight upregulation of inflammatory mediators, the profiles of which differed between the cell types studied. This attenuated effect, when compared to other cell types, most likely resulted from the low percentage of Ang II receptors in the fibroblast-like cells of the gingiva and periodontal ligament. Compared to Ang II, the IL1β challenge produced a robust upregulation of mRNA expression of inflammatory mediators without a synergistic effect with Ang II.

## Data Availability

The contents underlying the research text are contained in the manuscript.
